# Promising treatment biomarkers in asthma

**DOI:** 10.3389/fdsfr.2023.1291471

**Published:** 2023-11-07

**Authors:** Barbara Bonnesen, Jens-Ulrik S. Jensen, Alexander G. Mathioudakis, Alexandru Corlateanu, Pradeesh Sivapalan

**Affiliations:** ^1^ Department of Medicine, Section of Respiratory Medicine, Herlev-Gentofte Hospital, Hellerup, Denmark; ^2^ Department of Clinical Medicine, University of Copenhagen, Copenhagen, Denmark; ^3^ Division of Immunology, Immunity to Infection and Respiratory Medicine, School of Biological Sciences, The University of Manchester, Manchester, United Kingdom; ^4^ North West Lung Centre, Wythenshawe Hospital, Manchester University NHS Foundation Trust, Manchester Academic Health Science Centre, Manchester, United Kingdom; ^5^ Department of Respiratory Medicine, Nicolae Testemiţanu State University of Medicine and Pharmacy, Chisinau, Moldova

**Keywords:** asthma, biomarker, phenotype, personalized medicine, asthma treatment

## Abstract

Asthma is a highly heterogenous disease which researchers over time have attempted to classify into different phenotypes and endotypes to improve diagnosis, prognosis and treatment. Earlier classifications based on reaction to environmental allergens, age, sex and lung function have evolved, and today, the use of precision medicine guided by biomarkers offers new perspectives on asthma management. Identifying biomarkers that may reveal the underlying pathophysiology of the disease will help to select the patients who will benefit most from specific treatments. This review explores the classification of asthma phenotypes and focuses on the most recent advances in using biomarkers to guide treatment.

## 1 Introduction

Asthma is a chronic disease of the lower airways characterized by wheezing and airflow obstruction (Schoettler and Strek, 2020). It is estimated that 300 million people worldwide have asthma, placing considerable burdens on healthcare systems and economy (Loftus and Wise, 2015). Presently, the management of asthma revolves around symptom control, exacerbation prevention, and averting long-term complications. For mild to moderate asthma, standard treatment mainly consists of inhaled corticosteroids (ICS), long-acting beta-agonists (LABAs) and leukotriene antagonists, as recommended by the Global Initiative for Asthma (GINA) guidelines (Asthma, 2022).

According to GINA guidelines 3.7% of asthma patients have severe asthma, and ICS and LABA therapy has been proven insufficient (Asthma, 2022). In these cases, biologic treatment with monoclonal antibodies targeting specific cytokine pathway may be considered for selected phenotypes (Castillo et al., 2017), or additional treatment may encompass long-acting muscarinic-antagonists (LAMAs) and in some cases, azithromycin, an antibiotic known for its potential anti-inflammatory properties and impact on airway infections (Asthma, 2022). It is essential to recognize that not all asthma phenotypes respond favourably to the currently available treatment strategies, including biologic treatments, LAMA and azithromycin. Therefore, focus has shifted towards personalized medicine, highlighting the significance of biomarkers in directing the allocation of more costly treatment alternatives to patients that will benefit most from them.

This review will explore the phenotyping of asthma and focus on the biomarkers that may help guide treatment for patients with severe, uncontrolled asthma, Table 1.

**TABLE 1:** Overview of biomarkers in different asthma phenotypes and association to treatment.

Biomarker	Associated asthma phenotype(s)	Associated with effect of treatment with/useful to guide treatment with
FeNO	T2 high asthma Wenzel et al. (2013); Busse et al. (2015); Pavord et al. (2017); Shrestha et al. (2018); Takahashi et al. (2019); Akar-Ghibri et al. (2020); Wechsler et al. (2021)	ICS Tsurikisawa et al. (2012); Malin et al. (2014); Honkoop et al. (2015); Truong-Thanh et al. (2020); Heaney et al. (2021); Sutherland et al. (2021); Krishnan et al. (2022)
		OCS Kupczyk et al. (2013)
		Anti-IL-5 treatment Yamada et al. (2021)
		Anti-IL-4 and IL-13 treatment Castro et al. (2018); Corren et al. (2021a); Pavord et al. (2023)
		Anti-IL-13 treatment Brightling et al. (2015); Panettieri et al. (2018); Gottlow et al. (2019)
		Anti-IgE treatment Loureiro et al. (2018)
		Anti-TSLP treatment Corren et al. (2021b); Menzies-Gow et al. (2021)
Blood eosinophils	T2 high asthma Fahy, (2015); Fowler et al. (2015); Molfino et al. (2016); Takahashi et al. (2019); Wechsler et al. (2021)	ICS Pavord et al. (2020); Heaney et al. (2021); Krishnan et al. (2022)
	Patients with frequent exacerbations and severe asthma Pavord et al. (2020)	Anti-IL-5 treatment Ortega et al. (2014a); Harrison et al. (2020); Chen et al. (2023)
		Anti-IL-4 and IL-13 treatment Castro et al. (2018); Corren et al. (2021a); Pavord et al. (2023)
		Anti-IL-13 treatment Hanania et al. (2016)
		Anti-IgE treatment Loureiro et al. (2018)
		Anti-TSLP treatment Corren et al. (2021b); Menzies-Gow et al. (2021)
		Azithromycin Brusselle et al. (2013) (low eosinophile counts)
Sputum eosinophils	T2 high asthma Fowler et al. (2015); Akar-Ghibri et al. (2020)	ICS Krishnan et al. (2022)
		OCS Kupczyk et al. (2013); Berthon et al. (2017)
		Anti-IL-4 and IL-13 treatment Castro et al. (2018); Corren et al. (2021a); Pavord et al. (2023)
Blood neutrophils	Patients with frequent exacerbations and severe asthma Rhyou and Nam, (2020)	
Th17 cells	T2 low asthma Robinson et al. (2017)	
Total IgE	T2 high asthma Wenzel et al. (2013); Busse et al. (2015); Shrestha et al. (2018); Kuruvilla et al. (2019); Wechsler et al. (2021)	Anti-IL-5 treatment Yamada et al. (2021)
Specific IgE	Allergic asthma Romanet-Manent et al. (2002)	
IL-1β	T2 low asthma Liu et al. (2019)	
IL-4	T2 high asthma Wenzel et al. (2013); Kuruvilla et al. (2019); Akar-Ghibri et al. (2020)	
IL-5	T2 high asthma Ayars et al. (2013); Kuruvilla et al. (2019); Akar-Ghibri et al. (2020)	Anti-TSLP treatment Corren et al. (2021b); Menzies-Gow et al. (2021)
IL-6	T2 low asthma Liu et al. (2019)	
	Obesity associated asthma Dixon et al. (2006)	
	Patients with frequent exacerbations and severe asthma Peters et al. (2020); Sparreman Mikus et al. (2022)	
IL-10		ICS Tsurikisawa et al. (2018)
IL-13	T2 high asthma Wenzel et al. (2013); Scheerens et al. (2014); Kuruvilla et al. (2019); Akar-Ghibri et al. (2020)	ICS Tsurikisawa et al. (2018)
		Anti-TSLP treatment Corren et al. (2021b); Menzies-Gow et al. (2021)
IL-14		Anti-IgE treatment Loureiro et al. (2018)
IL-17A	T2 low asthma Robinson et al. (2017)	
IL-17F		
IL-21		
IL-22		
IL-25	T2 high asthma Kuruvilla et al. (2019)	
IL-33	T2 high asthma Kuruvilla et al. (2019); Wechsler et al. (2021)	
Periostin	T2 high asthma Fahy, (2015); Takahashi et al. (2019); Akar-Ghibri et al. (2020)	ICS Hoshino et al. (2016); Solanki et al. (2019); Heaney et al. (2021)
		Anti-IL-13 treatment Brightling et al. (2015); Hanania et al. (2016); Panettieri et al. (2018); Gottlow et al. (2019)
		Anti-IgE treatment Loureiro et al. (2018)
		Anti-TSLP treatment Corren et al. (2021b); Menzies-Gow et al. (2021)
TSLP	T2 high asthma Kuruvilla et al. (2019)	Anti-TSLP treatment Corren et al. (2021b); Menzies-Gow et al. (2021)
Chemokine ligand 13	T2 high asthma Scheerens et al. (2014)	
Chemokine ligand 17		
hs-CRP	Obesity associated asthma Dixon et al. (2006)	
GM-CSF	T2 high asthma Molfino et al. (2016)	
Acetylcholin		ICS Tsurikisawa et al. (2012)
TARC	T2 high asthma Wenzel et al. (2013)	
Eotaxin-3	T2 high asthma Wenzel et al. (2013); Wechsler et al. (2021)	
PARC	T2 high asthma Wechsler et al. (2021)	
YKL-40	T2 low asthma Sun et al. (2020)	
PAI-1	Obesity associated asthma Leija-Martinez et al. (2021)	
RBP4		
Mast cell carboxypeptidase A gene expression		OCS Berthon et al. (2017)
Alpha-1-antichymotrypsin	Patients with frequent exacerbations and severe asthma Sparreman Mikus et al. (2022)	
Apolipoprotein-E Complement factor I		
Complement component 9		
Glutathione S-transferase		
Macrophage inflammatory protein-3		
Sphingomyelin phosphodiesterase 3		
TGF-β		
TNFR11a		
Sputum IL-1B	Patients with frequent exacerbations and severe asthma Fricker et al. (2019)	
Sputum TNFR1		
Sputum TNFR2		
Sputum CXCR2		
Sputum Charcot–Leyden crystal galectin		
Sputum carboxypeptidase 3		
Sputum deoxyribonuclease 1-like 3		
Sputum alkaline phosphatase		
Sputum hyaluronan	T2 high asthma Ayars et al. (2013)	
More diverse airway microbiota	T2 high asthma Taylor et al. (2018)	
Less diverse airway microbiota	Neutrophilic asthma Taylor et al. (2018)	

Immunoglobulin E (IgE) Interleukin (IL) Fractional exhaled nitric oxide (FeNO) Thymic stromal lymphopoietin (TSLP) Thymus and activation-regulated chemokine (TARC) Activation-regulated chemokine (PARC) G ranulocyte-macrophage colony-stimulating factor (GM-CSF)T umour necrosis factor receptor (TNFR) High sensitive C-reactive protein (hs-CRP) Retinol-binding protein (RBP4)P lasminogen activator inhibitor (PAI-1) C-X-C motif chemokine receptor (CXCR)Transforming Growth Factor -β (TGF-β)Inhaled Corticosteroid (ICS) Oral Corticosteroid (OCS).

## 2 Asthma phenotypes

Asthma has been divided into allergic and non-allergic phenotypes. Allergic or atopic asthma is characterized by an earlier onset, and the reactions towards environmental allergens contribute to the development of symptoms and airway inflammation (Schatz and Rosenwasser, 2014; Akar-Ghibri et al., 2020). It can be diagnosed by relevant response to exposure to various allergens combined with antigen-specific tests with increased immunoglobulin E (IgE) (Romanet-Manent et al., 2002). Allergic asthma is more frequently associated with atopic dermatitis or eczema, conjunctivitis and possibly rhinitis (Romanet-Manent et al., 2002; Schatz and Rosenwasser, 2014; Akar-Ghibri et al., 2020), and it is associated with increased T2 inflammation and higher levels of cytokines such as interleukin (IL)-4, IL-5 and IL-13. T2 inflammation is also associated with increased levels of blood and sputum eosinophils, fractional exhaled nitric oxide (FeNO) and the lesser known periostin (Akar-Ghibri et al., 2020). Non-allergic or type 2 low asthma is a heterogenous phenotype, without clear cut definition, less well-characterised biomarkers and fewer, less effective treatment options.

Stratification of asthma into phenotypes can be based on clinical features, genetics, proteomics and metabolomics, as well as by biomarkers. The area is complex and the feasibility of obtaining sample in a clinical everyday setting varies, however proteomics (Riccio et al., 2020; Suzuki et al., 2021) and metabolomics (Maniscalco et al., 2018; Kachroo et al., 2021) definitely play a huge role in future asthma phenotyping, and a study even points to a role of lipidomic profiling (Brandsma et al., 2023).

Microbiota composition may also vary among patients with different asthma phenotypes. One study examined 167 participants with various (eosinophilic, neutrophilic, paucigranulocytic, or mixed) asthma phenotypes and found that airway microbiology was less diverse and more dissimilar in participants with neutrophilic asthma than in those who had eosinophilic asthma (Taylor et al., 2018).

Surprisingly, the pathophysiological asthma phenotypes may not be lifelong, or even long term. In a cohort study of 169 patients, asthma phenotype clusters were defined by physiological variables (i.e., lung function, reversibility and age of onset of the disease) or by biomarkers (i.e., FeNO levels and differential counts in induced sputum). Patients were followed up after 1 year, and at this time, the cluster allocation of a large proportion of the patients had changed (in 24% of patients stratified by physiological variables and 42% of patients stratified by FeNO levels and differential counts in induced sputum). The variability of the phenotypes was not influenced by change in oral or inhaled corticosteroid dose, nor by the number of exacerbations (Kupczyk et al., 2014).

### 2.1 T2 high asthma

T2 high asthma is characterized by reduced lung function, poor asthma control, increased IgE levels, increased responsiveness to ICS treatment and persistent eosinophilic inflammation mediated by cytokines such as IL-4, IL-5 and IL-13. The inflammation is aggravated by activation of thymic stromal lymphopoietin (TSLP), IL-25 and IL-33 as a reaction to microbes and allergens. This activates T cells, B cells and innate lymphoid cells, which in turn produce the cytokines involved in T2 high asthma. (Kuruvilla et al., 2019).

Patients with T2 high asthma are frequently atopic, more likely to be young at diagnosis and less likely to have a high body mass index (BMI). They frequently exhibited high bronchodilator reversibility (≥20%) in a retrospective analysis of 698 patients with poorly controlled asthma. (Busse et al., 2015; Shrestha et al., 2018).

FeNO is an easily measured indicator of T2 airway inflammation because it is produced by nitric oxide synthase in the airway epithelium, which is induced by IL-13 (Kuo et al., 2019). The U-BIOPRED study on 610 patients with asthma found that increased levels of FeNO could predict and classify T2 high asthma (Busse et al., 2015; Pavord et al., 2017; Shrestha et al., 2018), and high FeNO levels have been linked to asthma attacks (Couillard et al., 2021), however FeNO did not correlate with sputum eosinophilia in a study on 328 patients with asthma (Hastie et al., 2013).

The T2 high-associated increase in FeNO levels could be decreased by combined inhibition of IL-4 and IL-13 signalling in 104 patients with persistent, moderate-to-severe asthma. The decrease in FeNO correlated well with an improvement in Forced Expired Volume in the first second (FEV_1_) (Wenzel et al., 2013). Inhibition of the upstream alarmin IL-33 using a monoclonal antibody also reduced FeNO levels in 296 patients with moderate-to-severe asthma (Wechsler et al., 2021).

High IgE levels may also be associated to T2 high asthma (Busse et al., 2015; Shrestha et al., 2018), and like FeNO the T2 high-associated increased total IgE levels decreased during inhibition of alarmin IL-33 (Wechsler et al., 2021), IL-13 (Scheerens et al., 2014) and dual IL-4 and IL-13 signalling (Wenzel et al., 2013).

Blood eosinophilia and sputum eosinophilia are intrinsic to T2 high asthma, but do not correlate in all studies; in a study based on data collected from a clinical database of 163 patients attending the Manchester Severe Asthma Service between 2006 and 2013 found that using a cut-off of 0.45 × 10^9^ cells/L for blood eosinophilia could predict airway eosinophilia in patients with severe asthma (Fowler et al., 2015), and another study found that both blood eosinophil counts and serum periostin levels were good indicators of T2 high asthma and could help to guide treatment with personalized medicine (Fahy, 2015). However, in the study on 328 patients with asthma with no correlation between FeNO and sputum eosinophiles, there was also no correlation between blood eosinophil counts and sputum eosinophil levels (Hastie et al., 2013). Inhibition of IL-13 yielded no reduction in airway eosinophils in 64 patients with uncontrolled asthma (Austin et al., 2020), however inhibition of alarmin IL-33 reduced blood eosinophil counts in 296 patients with moderate-to-severe asthma (Wechsler et al., 2021).

Periostin is an extracellular matrix protein with an upregulated expression in airway epithelial cells and fibroblasts after activation by IL-13 and alarmin IL-33 (Wechsler et al., 2021) in patients with some types of asthma and allergic inflammation, but also in pulmonary fibrosis (Murata et al., 2018; Nukui et al., 2019; Katoh et al., 2020; Matsumoto, 2020). It may be linked to T2 high inflammation and correlate to type 2 asthma markers such as FeNO and blood eosinophils (Takahashi et al., 2019) but not necessarily sputum eosinophils (Cianchetti et al., 2019), and it may be suppressed by ICS (Solanki et al., 2019). In the context of activation in pulmonary fibrosis, periostin expression is also associated to persistent airway obstruction (FEV_1_/vital capacity [VC] < 88%) (Cianchetti et al., 2019).

Many other potential biomarkers have been investigated in RCTs involving patients with T2 high asthma. Many proteins are activated or inhibited by interleukins associated with type 2 asthma. For example, sputum hyaluronan (a matrix glycosaminoglycan) levels may be decreased by inhibiting IL-5 (Ayars et al., 2013), inhibiting IL-13 signalling decreases the levels of chemokine ligands 13 and 17 by approximately 25% (Scheerens et al., 2014), and inhibiting IL-4 and IL-13 signalling lowers thymus and activation-regulated chemokine (TARC) and eotaxin-3 levels (Wenzel et al., 2013). Inhibiting IL-33 reduced plasma eotaxin-3 as well as serum pulmonary and activation-regulated chemokine (PARC) levels (Wechsler et al., 2021). Higher levels of transforming growth factor (TGF)-beta, and lower levels of granulocyte-macrophage colony-stimulating factor (GM-CSF) and basic fibroblast growth factor (bFGF) was associated to persistent airway obstruction (FEV_1_/VC < 88%) in asthma (Cianchetti et al., 2019), but it is unknown, if they can predict it. An RCT examined the effect of inhibiting GM-CSF, which benefitted especially patients with blood eosinophils >300/mL or FEV_1_ reversibility ≥20% (Molfino et al., 2016). All these possible future biomarkers need further studies before clinical use.

Other proteins seem less promising as biomarkers; though OX40/OX40L interaction contributes to the allergic T cell response and helps sustain memory T cells, inhibition of the OX40/OX40L had no effect on airway hyperresponsiveness or blood eosinophil counts (Gauvreau et al., 2014a). Similarly, no association between IL-8 and RANTES levels and persistent airway obstruction was observed (Cianchetti et al., 2019), and hence neither of these are promising biomarkers to pursue.

Aspirin-induced asthma may pose a challenge for only 0.5% of the population, however patients with aspirin-induced asthma were more likely to take sick leave, require emergency treatment for asthma or experience lower respiratory symptoms than patients with aspirin-tolerant asthma in a Swedish questionnaire of 30,000 randomly selected subjects. Obesity is a strong risk factor for aspirin-induced asthma, traditionally making it challenging to treat, (Eriksson et al., 2015), however a recommended treatment is aspirin desensitization, though it may be associated with adverse reactions. A tiny RCT examined treatment with IgE inhibition prior to desensitization with possible effect, however it included only 11 participants (Lang et al., 2018). Hence, IgE signalling may be involved in aspirin-exacerbated respiratory disease and may have a role to play as a biomarker for prophylactic treatment before desensitization therapy.

### 2.2 T2 low asthma

T2 low asthma is a polymorphic entity characterized by severe, uncontrolled asthma in absence of T2 inflammation. Patients with type 2 low asthma had more asthma primary care attendances, were more likely to have a previous admission to an ICU and to be receiving maintenance oral corticosteroids in a study on 301 patients (McDowell et al., 2022).

There are biomarkers useful for various phenotypes of non-eosinophilic and T2 low asthma; Th17 cells may be responsible for associated with a severe asthma phenotype, which is characterized by neutrophilic inflammation and interleukins such as IL-17A, IL-17F, IL-21 and IL-22 (Robinson et al., 2017). Neutrophilic inflammation may also be associated with YKL-40 in serum and in lung tissue, as it is secreted by neutrophils, and correlates with increased severity of asthma (Sun et al., 2020), and with cytokines such as IL-1β and IL-6 (Liu et al., 2019).

An analysis based on the AMAZES RCT with 142 patients, showed that in the sputum of patients with neutrophilic asthma tumour necrosis factor receptor (TNFR)1 and TNFR2 were significantly increased with a dose-response relation to more severe neutrophilic asthma and correlated with poorer lung function, poorer asthma control, and increasing age (Niessen et al., 2021).

### 2.3 Obesity associated asthma

Asthma symptoms such as wheezing and shortness of breath are more prevalent among people with elevated BMI in a study involving 85,437 Danes using Mendelian randomization (Colak et al., 2016) and a cross-sectional survey of 15,454 Europeans (Jarvis et al., 2002). The degree of obesity has been shown to be negatively correlated with lung function (Huang et al., 2012), and a small RCT with 90 patients showed that abdominal adiposity was associated with poorer asthma control, even after controlling for BMI (Lv et al., 2014). Therefore, asthma control and asthmatic symptoms may be associated with both obesity and distribution of adiposity.

Obesity has been shown to be associated with elevated levels of pro-inflammatory cytokines; IL-6 and highly sensitive assays for serum C-reactive protein (hs-CRP) from <2 to >7 mg/mL (Dixon et al., 2006). A study on asthma in adolescents investigated retinol-binding protein (RBP4) and plasminogen activator inhibitor (PAI-1) and found that RBP4 was useful in predicting non-allergic asthma in adolescents with obesity (Leija-Martinez et al., 2021). Therefore, RBP4 may be useful in identifying obesity-related asthma, differentiating it from other forms of asthma. However, further research is needed.

T2 high symptoms and biomarkers have also been investigated in obese patients with suspected T2 high asthma. Obesity was not associated with hayfever or nasal allergies, or with levels of IgE to house dust mites, grass, or cats, or with total IgE levels in the cross-sectional survey of 15,454 Europeans (Jarvis et al., 2002). Similarly, sinonasal disease was not associated with obesity in an RCT of 236 patients with asthma (Kanagalingam et al.).

### 2.4 Patients with frequent exacerbations and severe asthma

A simple history-based model extended with spirometry could identify patients who developed asthma exacerbations in a primary care-based cohort analysis of 611 patients. The additional value of FeNO was modest; however, the patients in this study were not phenotyped, which may have masked a potential predictive role of FeNO among patients with T2 high disease (Loymans et al., 2016). Another primary care based RCT, also with patients who were not phenotyped, studied the risk of exacerbations among 304 patients followed for 12 months. This study highlighted some parameters that may be used to quantify future risk: female gender (OR, 2.34), a severe exacerbation in the previous year (OR, 3.17), an increase of half a point on the baseline Asthma Control Questionnaire (ACQ) (OR, 1.87), and an additional litre in FEV_1_ (OR, 0.63); however, there was no association with FeNO levels (Boer et al., 2019).

Initial studies on asthma showed that the risk of a severe asthma event increased in patients who were undergoing treatment with short-acting beta-agonists (SABAs) alone. However, in an RCT with 675 patients who were followed for 52 weeks, the proportion of patients undergoing treatment with SABAs alone who had a severe exacerbation increased progressively with increasing blood eosinophil count (Pavord et al., 2020). Among patients receiving moderate dose ICS, exacerbations were associated with lower eosinophil counts in blood and sputum and with higher neutrophil counts in sputum (Rhyou and Nam, 2020). Hence, blood and sputum eosinophil counts may be good biomarkers for ICS treatment suitability and for quantifying the risk of exacerbations without ICS treatment, whereas sputum neutrophils may be a marker for a high risk of exacerbations despite ICS treatment.

One study investigated potential sputum biomarkers in a cohort of 142 patients from the AMAZES RCT with asthmatic symptoms despite ICS and LABA treatment. It defined sputum Charcot–Leyden crystal galectin, carboxypeptidase 3, deoxyribonuclease 1-like 3, alkaline phosphatase, C-X-C motif chemokine receptor (CXCR)2, and IL-1B as possible predictors of an exacerbation. (Fricker et al., 2019). Although CXCR2 may predict exacerbations among symptomatic patients despite ICS and LABA treatment, an antagonist of CXCR2 (AZD5069) was not a promising target for therapy in a phase III RCT; among 640 patients of whom 478 received AZD5069, there was no effect on the primary endpoint of frequency of severe exacerbations (O'Byrne et al., 2016).

Another RCT, involving 147 patients, investigated the effect of inhaled interferon (IFN)-beta as a treatment for asthma exacerbations following cold symptoms to assess the risk of exacerbations after a viral infection. The study found that inhaled IFN-beta had no effect on ACQ, but there was an effect on secondary endpoints including peak expiratory flow recovery, the need for additional treatment, and boosted innate immunity as assessed by blood and sputum biomarkers (Djukanovic et al., 2014). A study of 406 asthmatic patients found that IL-6 levels were correlated with frequent exacerbations, whereas blood eosinophil counts were not (Peters et al., 2020).

A biomarker study on 142 patients with mild-moderate and severe asthma, showed that elevated levels of alpha-1-antichymotrypsin, apolipoprotein-E, complement component 9, complement factor I, macrophage inflammatory protein-3, IL-6, sphingomyelin phosphodiesterase 3, TNF receptor superfamily member 11a, TGF-β and glutathione S-transferase was associated to more severe asthma. Treatment with oral corticosteroids decreased the expression of most, but not all of these biomarkers (Sparreman Mikus et al., 2022).

### 2.5 Treatment response

Another method for identifying severe asthma phenotypes involves studying the response to different treatments and then retrospectively determining the characteristics of patients’ asthma. One recent study used responsiveness to anti IL-5 medication as an indicator of different phenotypes of severe eosinophilic asthma by monitoring changes in FEV_1_ (Yamada et al., 2021). This study identified five phenotypes based on differences in the FEV_1_ response to medication and the Asthma Control Test (ACT) response to medication. Further, there were differences in the quantities of type 2 inflammation markers, age of onset, smoking exposure and BMI. The most responsive cluster had a moderate increase in IgE and FeNO levels relative to blood eosinophils and had less exposure to smoking. The moderately responsive clusters had a greater degree of T2 inflammation, compared with the non-responsive clusters.

## 3 Precision medicine

The identification of different asthma phenotypes or endotypes can contribute to relevant treatment of patients as they provide an option for precision medicine targeted towards specific cellular signalling pathways based on specific biomarkers. Such medicine includes ICS, antibiotics, as well as biological treatment using monoclonal antibodies targeted against cellular signalling pathways based involved in the pathophysiology of asthma, for example, cytokines involved in Th2 inflammation in patients with asthma.

### 3.1 Inhaled corticosteroids

The effect of ICS may be predicted using patient clinical characteristics and biomarkers. Low and normal BMI predicted a therapeutic effect of ICS in a *post hoc* analysis of 894 patients with moderate asthma in pooled data from four double-blind, placebo controlled RCTs. Patients were treated with beclomethasone; however, the effect of beclomethasone decreased with increasing BMI, and was only significant among patients with low or normal BMI (Peters-Golden et al., 2006).

Sputum and blood eosinophiles as well as FeNO predicted response to ICS treatment in an RCT on 237 patients with uncontrolled mild-moderate persistent asthma, (Krishnan et al., 2022). In the BASALT RCT FeNO based adjustment of ICS treatment yielded no significant differences in time to treatment failure. This RCT from 2012 examined 342 patients allocated to three strategies for optimising low-dose ICS therapy: physician assessment-based adjustment, FeNO based adjustment, and symptom-based adjustment for 9 months follow-up with adjustments every 6 weeks for FeNO and physician assessment-based treatment. (Calhoun et al., 2012). One weakness of the BASALT RCT was that the patients were not included by or divided into groups based on phenotypes, which may have masked the benefits of using FeNO as a biomarker for ICS treatment. In a retrospective cohort study of 153 non-smoking, steroid-naïve patients initial FeNO level of ≥25 ppb was associated to an improvement in ACQ during treatment with 500 µg beclomethasone for 6 weeks (Malin et al., 2014). Another cohort study has document that FeNO often decreases with ICS treatment; in a cohort study of 102 Tamilian patients, FeNO decreased from 91–103 ppb to 75–90 ppb over 8 weeks of treatment with ICS (Neelamegan et al., 2016). Hence, despite it frequent use in clinical practise, the role of FeNO as a biomarker is not clear-cut.

In a cohort study of 40 patients with mild-to-moderate asthma, serum periostin decreased from 90 to 59 ng/mL during 4 weeks of ICS treatment, which correlated with an increase in peak expiratory flow rate (68 L/min) and in Asthma QOL (Solanki et al., 2019). Similarly, 42 healthy control subjects and 20 previously ICS naïve patients with asthma who were treated with ICS exhibited decreases in serum periostin, correlating with increased FEV_1_%, decreased sputum eosinophils and decreased bronchial wall area (Hoshino et al., 2016). Hence, periostin may have a potential as a biomarker for ICS treatment efficacy, at least in some asthma phenotypes. However, these are preliminary data.

In a very small study of 23 patients with steroid resistant asthma, IL-17A and IFN-γ were elevated in comparison to patients with steroid sensitive asthma (Chambers et al., 2015). However, whether these interleukins would be suitable biomarkers remains unclear.

In conclusion, ICS treatment may be particularly beneficial for patients with asthma who have low or normal BMI, elevated sputum eosinophils and/or elevated periostin, and possibly elevated FeNO levels.

### 3.2 Corticosteroid reduction

Corticosteroid dose may be reduced in accordance with different strategies for monitoring asthma control and treatment efficacy; a lower daily ICS dose (375 ± 203 vs. 424 ± 221 mcg) was achieved at 9 months follow-up among patients whose treatment was guided by Global Initiative for Asthma (GINA) guidelines and FeNO levels, compared with patients whose treatment was guided by GINA alone in an RCT of 176 symptomatic patients with asthma (Truong-Thanh et al., 2020). Similarly, a strategy involving treatment guided by ACQ and FeNO yielded improved asthma control and a lower accumulated dose of asthma medication but not increased quality of life, compared with treatment guided by ACQ alone in a nonblind cluster-randomized trial of 611 patients in primary care (Honkoop et al., 2015). Finally, in a Japanese cohort study of 90 patients with moderate or severe asthma, a successful reduction in ICS dose of 50% was achieved guided by FeNO levels (Tsurikisawa et al., 2012). However, one weakness common to these three studies is that the patients were not phenotyped before study entry. Different asthma phenotypes may generate different results, and different monitoring algorithms may be more relevant for particular groups of patients.

In the LASST RCT, a large proportion of the 447 patients had T2 high disease. The patients were treated with moderate doses of ICS, subjected to a step-down algorithm, and followed for 56 weeks. During the trial, FeNO was measured at 6-week intervals, as well as other biomarkers, including levels of multiallergen IgE. None of these biomarkers, including FeNO, predicted loss of asthma control during the ICS step-down process (Bose et al., 2020).

To investigate patients with T2 low asthma, an RCT examined 180 adults with suspected asthma and FeNO ≤27 ppb. In this study, a daily dose of 400 µg budesonide had no effect on symptom control or on FEV_1_ (Sutherland et al., 2021). Hence, in patients with asthma symptoms and low FeNO levels, ICS may have limited benefit and dose may be decreased with little risk of lowering symptom control or quality of life.

Using a more extensive algorithm that included blood eosinophil counts, serum periostin and FeNO levels also led to a greater decrease in corticosteroid dose than treatment guided by asthma symptoms, lung function and recent exacerbation history. This was achieved without a drop in asthma control or quality of life in a single-blind, parallel group RCT of 301 patients with severe asthma, who were treated based on the algorithm for 48 weeks. In this study, an inclusion criterion was FeNO <45 ppb (Heaney et al., 2021). Therefore surprisingly, a T2 high-based algorithm was effective in predicting safe ICS reduction in patients with T2 low asthma. However, in a *post hoc* analysis of this trial the decrease in corticosteroid dose was only seen in women (Eastwood et al., 2023).

Blood eosinophil counts ≥0.3 × 10^9^/L were able to predict an effect of ICS in an open-label RCT of 675 patients with mild asthma. Additionally, in this trial, 200 µg of budesonide twice a day was not more effective than SABA alone in preventing exacerbations among patients with blood eosinophil counts <0.15 × 10^9^/L during a 52-week follow-up period (Pavord et al., 2020).

In a Japanese cohort study, low serum IL-33 levels, low peak expiratory flow variability over 1 week, childhood onset of asthma (at age <10 years), and high serum IL-10 levels were associated with a safe reduction in ICS dose. In this cohort study, 223 patients underwent a 50% reduction in ICS dose over 36 months and were monitored for asthma control (Tsurikisawa et al., 2018). In another Japanese cohort of 90 patients with a similar setup, the predictors of a safe 50% reduction in ICS dose were serum acetylcholine levels, FeNO levels, length of time with no clinical symptoms before dose reduction, as well as higher FEV_1_% (Tsurikisawa et al., 2012). Hence, many of these parameters are potential clinical markers and biomarkers for ICS treatment suitability and/or safe ICS dose reduction. However, appropriate trials are needed before they can be implemented clinically.

Finally clinical parameters such as poor adherence, chronic upper airway complications and, to a lesser degree, sleep disorders and psychiatric comorbidities, such as depression, anxiety and somatoform disorders have been associated with failed ICS dose reduction in a cohort of 222 well-controlled asthma patients receiving ICS or ICS/LABA (Saito et al., 2017).

In conclusion, ICS dose may be reduced safely in some patients with low FeNO levels, blood eosinophil counts <0.15 × 10^9^/L, low IL-33 levels, high IL-10 levels, high serum acetylcholine, low peak expiratory flow variability, and/or a long period of time with no clinical symptoms.

### 3.3 Avoiding oral steroid treatment

An RCT investigated the effect of treatment with 50 mg prednisolone for 10 days in patients with stable asthma. Patients with higher levels of sputum and blood eosinophils, higher levels of mast cell carboxypeptidase A gene expression, or poorer lung function were more likely to have a beneficial response to treatment with oral corticosteroids (Berthon et al., 2017).

Another RCT examined treatment with 0.5 mg/kg prednisolone for 2 weeks in 84 patients with severe asthma and 62 patients with mild-to-moderate asthma. The patients who experienced beneficial effects from treatment with oral corticosteroids had higher FeNO levels, higher levels of sputum eosinophils, lower FEV_1_% and lower quality of life (Kupczyk et al., 2013).

Hence, patients with T2 high asthma or with poor lung function may experience greater benefits from short-term, oral corticosteroid treatment than patients with T2 low asthma.

### 3.4 Montelukast

Montelukast is an inhibitor of [3H]-leukotriene D4-receptor specific binding (Schoors et al., 1995). In a *post hoc* analysis with pooled data from four RCTs including 3,073 patients with moderate asthma montelukast was as effective for patients with obesity as it was for patients with normal BMI. In contrast, the effectiveness of beclomethasone decreased with increasing BMI (Peters-Golden et al., 2006). Hence, montelukast might be a more attractive treatment option for patients with asthma and obesity. Additionally, montelukast might benefit patients with asthma and chronic rhinosinusitis with nasal polyps in combination with nasal corticosteroid treatment and surgery (Nonaka et al., 2010), however there is no data on the predictive value of nasal involvement in treatment of asthma with montelukast.

### 3.5 Roflumilast

Roflumilast is an oral, once-daily phosphodiesterase 4 inhibitor that was developed to treat chronic obstructive pulmonary disease (COPD) and asthma. It decreases the levels of tumour necrosis factor (TNF)-alpha (Timmer et al., 2002).

A meta-analysis of evidence from eight placebo-controlled, double-blind phase I–III studies with a total of 197 patients examined the effect of roflumilast, compared to placebo. In all studies, the patients had asthma and the required pre-bronchodilator FEV_1_% was at least 50%; in five of the eight studies, and in the remaining the required pre-bronchodilator FEV_1_% was ≥70%. The meta-analysis found that roflumilast attenuated early and late allergen-induced bronchoconstriction and reduced FeNO levels, sputum eosinophil and neutrophil counts, and serum TNF-alpha and urinary leukotriene E4 levels (Bardin et al., 2015). An effect of Roflumilast on FEV_1_ was seen in a large noninferiority RCT of 499 patients with persistent asthma, in which roflumilast improved FEV_1_% by 12%. This was similar to the effect of ICS, which increased FEV_1_ by 14%. However, in the trial, the patients had an FEV_1_% of 50%–85%, indicating a potentially chronic or remodelling component to their obstructive disease (B et al., 2006). A small RCT examined combined treatment with roflumilast and montelukast and found a possible additive effect in FEV_1_% improvement, patient-reported outcomes, and a further reduction in urinary leukotriene E4 levels (Bateman et al., 2016).

Hence, roflumilast may have a role among asthmatics with chronically decreased FEV_1_.

### 3.6 Anti-IL-5 treatment: mepolizumab, reslizumab and benralizumab

Anti-IL-5 treatment by specific blocking antibodies is available as benralizumab, mepolizumab and reslizumab. This treatment leads to depletion of eosinophils and reduction of eosinophilic inflammation (Brusselle and Koppelman, 2022).

Mepolizumab reduces exacerbation rates and the need for oral corticosteroids, and it improves asthma control, quality of life and lung function in patients with severe asthma and elevated blood eosinophils (≥300 cells/µL) (Ortega H. G. et al., 2014; Harrison et al., 2020). It also reduces blood eosinophil counts (Ortega H. G. et al., 2014). The DREAM RCT showed that the effects of mepolizumab were enhanced in patients with elevated blood eosinophils, and in those with higher airway reversibility (Ortega H. et al., 2014), and *post hoc* analyses of data from the DREAM and MENSA RCTs showed that the exacerbation rate reduction with mepolizumab increased progressively from 52% in patients with blood eosinophil count ≥150 cells/µL to 70% in patients with blood eosinophil count ≥500 cells/µL (Ortega et al., 2016). Other key predictors for treatment benefit for severe exacerbations included exacerbation history, baseline ACQ5 score and age, and those for symptom control included blood eosinophil count and presence of chronic rhinosinusitis with nasal polyps, which also benefitted from the treatment (Chen et al., 2023; Chupp et al., 2023; Liu et al., 2023). In the MAPLE trial on 27 patients treated with mepolizumab, addition of oral corticosteroids had no significant effect on symptoms or quality of life, but they did still improve small-airway obstruction and reduce T2 biomarkers (Yang et al., 2022). Surprisingly, among patients with high airway reversibility, a higher BMI also correlated with increased benefits of mepolizumab treatment (Ortega H. et al., 2014).

Similarly, trials on reslizumab showed reduced exacerbation rates in patients with high eosinophilia and poorly controlled, moderate-to-severe asthma (Castro et al., 2015). In addition, chronic rhinosinusitis with nasal polyps was also associated with a high responsive to anti-IL-5 treatment with reslizumab (Weinstein et al., 2019).

Finally, studies that used benralizumab to treat uncontrolled eosinophilic asthma showed reduced exacerbation rates and increased FEV_1_ (FitzGerald et al., 2016; Park et al., 2016). Sera from patients in phase I and IIa studies were analysed before and after treatment with benralizumab, and these analyses showed that benralizumab reduced blood eosinophil counts and serum eosinophil-derived neurotoxin, as well as eosinophil cationic protein relative to baseline. No changes were observed in TNF or IFN-γ levels, whereas IL-5, eotaxin/CCL11, and eotaxin-2/CCL24 levels increased (Pham et al., 2016). Likewise, benralizumab, exhibits a notable positive effect on chronic rhinosinusitis with nasal polyps (Tversky et al., 2021; Bachert et al., 2022).

Hence, blocking the IL-5 pathway may be a good treatment option for patients with asthma and elevated blood eosinophils when ICS does not control the disease. Other biomarkers may also be used, but blood eosinophils are easy to measure in clinical practice.

### 3.7 Anti-IL-4 and anti-IL-13 treatment: dupilumab inhibits IL-4 and IL-13, and tralokinumab and lebrikizumab inhibits IL-13

IL-13 is a central mediator of allergic asthma. IL-13 is released along with IL-4 by Th2 cells in response to allergens and leads to IgE isotype switching, airway hyperresponsiveness, recruitment of eosinophils, macrophage activation, increased permeability of the airway epithelium and increased mucus production (Ingram and Kraft, 2012). IL-4 is a part of the same pathway and increases airway inflammation via isotype switching of B-cells and allergen sensitization (Busse, 2019).

Dupilumab is a monoclonal antibody that binds to the IL-4 receptor, blocking IL-4 and IL-13 signalling. Dupilumab lowered total IgE and FeNO, but not blood eosinophile levels in 104 patients with persistent, moderate-to-severe T2 high asthma with blood eosinophils ≥300 cells/µL or sputum eosinophils ≥3%. The decrease in FeNO levels induced correlated to improvement in FEV_1_. (Wenzel et al., 2013). Dupilumab also significantly reduced severe exacerbations and improved FEV_1_ and asthma control in the QUEST trial on patients with type 2 high asthma with elevated FeNO, blood eosinophils ≥300 cells/µL or sputum eosinophils ≥3% (Castro et al., 2018; Corren et al., 2021a; Pavord et al., 2023). The effects of dupilumab were enhanced in patients with concomitant chronic rhinosinusitis (Busse et al., 2020; Maspero et al., 2020) and among patients with persisting airway obstruction (Hanania et al., 2023). Hence, the phenotype for which dupilumab is a suitable treatment option has been examined thoroughly. After 52-week of treatment, dupilumab decreased FeNO levels, blood eosinophils, total IgE and TARC levels in an RCT on 1,902 patients (Busse et al., 2020; Maspero et al., 2020).

Lebrikizumab is an anti-IL-13 monoclonal antibody, which has been examined in two RCTs. The LAVOLTA I and II trials each continued for a total of 52 weeks and included 1,081 and 1,067 patients, respectively. Similar to dupilumab, lebrikizumab reduced exacerbation rates and improved FEV_1_ in patients with uncontrolled asthma. In particular, patients with high concentrations of type 2 biomarkers, such as serum periostin ≥50 ng/mL or blood eosinophils ≥300 cells/µL, benefitted from treatment with lebrikizumab (Hanania et al., 2016), just as with dupilumab.

Tralokinumab is another anti-IL-13 monoclonal antibody that was used to treat patients with severe, uncontrolled asthma in two phase III RCTs, STRATOS 1 and 2. Tralokinumab reduced asthma exacerbations in participants with severe asthma and FeNO ≥37 ppb in STRATOS 1, but not STRATOS 2. FeNO and periostin were the only biomarkers that could predict the effect of tralokinumab treatment (Brightling et al., 2015; Panettieri et al., 2018; Gottlow et al., 2019).

In conclusion, inhibition of IL-4 and IL-13, and to a lesser degree inhibition of IL-13 alone, may benefit patients with T2 high asthma, such as those with elevated FeNO levels, elevated blood eosinophils (≥300 cells/µL) or sputum eosinophils (≥3%), and/or elevated periostin. It may also benefit patients with concomitant chronic rhinosinusitis.

### 3.8 Anti-IgE treatment: omalizumab and quilizumab

Omalizumab binds IgE in the blood and interstitial fluid as well as membrane bound IgE on the surface of B lymphocytes. Omalizumab decreased asthma symptoms in patients with severe, uncontrolled asthma (*n* = 850) over 48 weeks (Hanania et al., 2011). In another study omalizumab might have benefitted patients with type 2 high asthma; allergic asthma and non-allergic asthma with high levels of FeNO, IL-14, eosinophils or periostin (Loureiro et al., 2018). Additionally, omalizumab has been proven beneficial for patients with chronic rhinosinusitis with nasal polyps (Damask et al., 2022; Gevaert et al., 2022), though neither comorbid allergic rhinoconjunctivitis, chronic rhinosinusitis, recurrent acute sinusitis nor nasal polyps was associated with the treatment response of omalizumab on asthma (Chen et al., 2021).

Quilizumab is a humanized IgG1 monoclonal antibody targeting IgE, leading to depletion of IgE-switched B-cells and memory B cells (Harris et al., 2016). It reduced serum IgE levels and attenuated the early and late asthmatic reaction following allergen challenge in 36 patients with mild asthma (Gauvreau et al., 2014b). Quilizumab reduced total-serum and allergen-specific IgE levels by 30%–40%, but had no impact on asthma exacerbations, lung function, or symptoms in an RCT on 578 patients with allergic asthma that could not be controlled by high-dose ICS (Harris et al., 2016).

In conclusion, anti-IgE treatment may have a role in particular subgroups of patients with type 2 high asthma or allergies, but no certain clinical markers or biomarkers that defines these subgroups has been identified.

### 3.9 Anti-thymic stromal lymphopoietin treatment: tezepelumab

TSLP is a member of the IL-2 cytokine family and is released from airway epithelia into the bronchoalveolar fluid as part of an allergic inflammatory response and may play a role in asthma pathogenesis that is primarily T2 driven (Li et al., 2018; Peebles and Aronica, 2019). Tezepelumab is an anti-TSLP human monoclonal antibody that reduced exacerbation rates, increased lung function, and improved asthma control and quality of life in RCTs (Corren et al., 2021b; Menzies-Gow et al., 2021; Corren et al., 2023).

Experience from the NAVIGATOR trial showed that Tezepelumab is more efficient among patients with allergies (Corren et al., 2023). The effects of tezepelumab were also greater in patients with T2 high asthma with higher levels of blood eosinophils, TSLP, FeNO, IL-5, IL-13 and serum periostin, and treatment with tezepelumab decreased the levelse of blood eosinophils, FeNO, IgE, IL-5, IL-13, periostin, TARC, and TSLP over a 52-week treatment period (Corren et al., 2021b; Menzies-Gow et al., 2021).

### 3.10 Antihistaminergic treatment

Histamine is released by mast cells and plays a role in regulating inflammatory processes in response to environmental allergens. These effects are mediated by T helper lymphocytes and cytokines such as IL-4, IL-10 and IL-13, which promote T2 high inflammation. Four histamine receptors are expressed in pulmonary tissue (Yamauchi et al., 2019); histamine receptor 1 (H1R) promotes cellular migration, vasodilatation and bronchoconstriction and plays a role in hypersensitivity reactions and allergic asthma. Activation of histamine receptor 4 (H4R) leads to increased expression of pro-inflammatory cytokines and migration of eosinophils (Thangam et al., 2018).

An oral H4R antagonist (JNJ-39758979) has been examined in a phase IIa RCT. The primary endpoint of improved FEV_1_% was not met. The study included 115 patients, who were followed during the 12-week trial. The study also found no clinically relevant changes in FeNO levels or blood or sputum eosinophil counts (Kollmeier et al., 2018).

### 3.11 Azithromycin

Azithromycin is a macrolide antibiotic that may be used to control asthma. In a planned *post hoc* analysis of an RCT, low-dose azithromycin was given to patients with severe asthma and blood eosinophils ≤200/µL. Azithromycin protected against severe exacerbations and lower respiratory tract infections requiring treatment with antibiotics (OR, 0.44). This effect was not seen in patients with eosinophilic asthma (OR, 1.03) (Brusselle et al., 2013). Similarly, azithromycin treatment was able to reduce sputum TNFR2 and TNF levels relative to placebo, but only in patients with non-eosinophilic asthma (Niessen et al., 2021). Hence, treatment with azithromycin may be particularly suitable for patients with type 2 low asthma. On the topic of chronic rhinosinusitis with nasal polyps, Azithromycin may (de Oliveira et al., 2020) or may not (Videler et al., 2011) be beneficial, however the data is insufficient to draw a conclusion, and there is no data on the predictive value of nasal involvement in treatment of asthma with azithromycin.

### 3.12 C-reactive protein, interleukin 6 and leptin

CRP is a marker of inflammation and infection that is synthesized in the liver (Sigari and Ghasri, 2013). It is regulated by IL-6, which is secreted locally in response to infection and tissue injury (Tanaka et al., 2014). A prospective case–control study of 100 patients with asthma and 50 healthy control subjects investigated the correlation between hs-CRP and spirometry, as well as for clinical indications of asthma control. The study found no correlation between hs-CRP and FEV_1_ or other indicators of asthma control such as ACT scores. hs-CRP was elevated in all patients with asthma who participated in the study, including those with controlled asthma (Sigari and Ghasri, 2013). In contrast, one study of 30 patients with asthma and 30 healthy control subjects reported that hs-CRP was related to severity of asthma and ACT scores, highlighting hs-CRP as a possible marker for the severity and control of asthma (Kilic et al., 2012). Another study of 120 patients and 115 control subjects measured hs-CRP and ACT scores, and found higher hs-CRP measurements in patients with asthma than in healthy control subjects, which increased with disease severity. (Monadi et al., 2016). Therefore, hs-CRP measurements may be useful in predicting asthma control and treatment response.

### 3.13 Long-acting muscarinic-antagonist treatment

FEV_1_ reversibility predicted a response to treatment with LAMA in an RCT of 237 patients with mild persistent asthma (Krishnan et al., 2022). LAMA treatment decreased airway wall thickness and improved airflow obstruction in an RCT that included 53 patients with symptomatic asthma and FEV_1_% 60%–90% who had previously been treated with ICS/LABA. Addition of LAMA did not affect FeNO levels (Hoshi et al., 2016). Hence, LAMA treatment may be suitable for the treatment of asthma in some patients, but further studies should investigate which phenotypes may benefit most from LAMA treatment.

### 3.14 Weight loss by diet and exercise

RCTs have suggested that a combination of weight loss and exercise may be effective in treating obesity-associated asthma. Weight loss and exercise improved asthma control and quality of life and decreased the use of reliever medication (Stenius-Aarniala et al., 2000; Scott et al., 2013; Dias-Junior et al., 2014; Ma et al., 2015; Scott et al., 2015; Freitas et al., 2017; Ozbey et al., 2020; Turk et al., 2020). A weight reduction of as little as 5%–10% may improve asthma control (Scott et al., 2013). However, these studies all had short-term (<12 months) follow-up periods (Stenius-Aarniala et al., 2000; Scott et al., 2013; Dias-Junior et al., 2014; Ma et al., 2015; Scott et al., 2015; Freitas et al., 2017; Ozbey et al., 2020; Turk et al., 2020). An RCT of 51 patients with a mixed intervention of weight loss and exercise and a 3 months follow-up period found effects on FEV_1_, FVC, and levels of FeNO, IL-4, IL-6, IL-10, leptin, adiponectin, TNF-alpha, and CCL2 (Freitas et al., 2017), some of which may serve as biomarkers for treatment with weight loss and exercise.

### 3.15 Weight loss by anorectics

The effects of anorectic medications, such as sibutramine (10 mg per day in patients with no cardiovascular risk factors) and orlistat (maximum dose of 120 mg per day), on asthma control in patients with obesity have also been investigated (Dias-Junior et al., 2014). Preliminary results show some promise; a study of 33 patients that investigated the effects of a calorie restricted diet and anorectics, with a 6 months follow-up period, showed that these interventions had an effect on asthma control, but no effects on sensitivity to methacholine challenge, FeNO levels, sputum counts, total IgE, or leptin, TGF-beta or eotaxin levels (Dias-Junior et al., 2014). A lot more research will be anticipated in this area with the arrival of Glucagon Like Peptide (GLP)-1 analogues.

### 3.16 Pulmonary rehabilitation

An RCT of 34 patients with severe obesity-associated asthma who underwent a 3-month high-intensity pulmonary rehabilitation programme found sustained improvements in asthma control 9 months after the intervention (Turk et al., 2020).

Hence, pulmonary rehabilitation may be very beneficial in patients with asthma, particularly obesity-associated asthma.

### 3.17 Proton pump inhibitors

An RCT that included outpatients with asthma and gastro-oesophageal reflux found that asthma symptoms were relieved or improved in 18 of 51 patients who had 40 mg of once-daily omeprazole for 8 weeks. Patients who responded well to treatment with proton pump inhibitors were more likely to have higher BMI or to have more severe reflux (Kiljander et al., 2001).

### 3.18 Probiotics

A small RCT with 36 patients looked at the effect of 2 months treatment with ataria multiflora Boiss (Z. multiflora) and saw a reduction in symptoms and an improvement in pulmonary function as well as a reduction in eosinophils and hs-CRP (Alavinezhad et al., 2022).

### 3.19 Procalcitonin for guiding use of antibiotics during exacerbations

Serum procalcitonin-guided antimicrobial therapy allows antibiotic exposure to be reduced in patients with severe acute asthma exacerbations, with no obvious adverse effects. This was confirmed by two RCTs of 169 and 293 patients requiring hospitalization for severe acute exacerbations of asthma. Serum procalcitonin-guided antimicrobial therapy was compared to standard antimicrobial therapy, and at 12-month follow-up, patients treated using the procalcitonin-guided antimicrobial therapy were substantially less likely to require antibiotic therapy. This was achieved without inferior outcomes in terms of clinical recovery, length of hospital stay, clinical, laboratory and spirometry measures (Tang et al., 2013; Long et al., 2014).

## 4 Barriers to the routine use of biomarkers

Although the phenotyping/endotyping of asthma and identification of relevant biomarkers provides opportunities to target treatments towards the underlying causes of the disease, there are also challenges and issues associated with the use of precision medicine via biomarkers. Some of these issues partly explain why biomarker measurements are not yet routinely used in clinical practice.

First, identifying patient phenotypes is time consuming and requires extra resources. Furthermore, reimbursement may not be as readily available for phenotyping as it is for other standard diagnostic tests. Second, some of the currently available biomarkers are measured from sputum samples, which may cause discomfort for patients, and requires specialist equipment and training, and the identification of phenotypes involves extra laboratory work. Third, there is a substantial gap in the available, RCT tested biomarkers used in everyday clinical life to assess relevant treatment options and treatment optimisation for patients with severe or difficult to treat asthma. This gap can only be closed by substantial research within the field, both in the areas of primary research and subsequent clinical testing in RCTs.

In some cases, asthma can remit to a less symptomatic and less severe disease level. In these cases it is important to focus on a therapeutical step down strategy. Focus on limiting oral corticosteroid treatment is already in practice, however step down of other side-effect prone therapeutics should also be prioritised. Even treatment with biologics may be reduced without recurrence of a severe asthma phenotype after a period with well-controlled disease. At present, we have very little biomarkers predictive of remission. Some biomarkers may aide in determining a relevant ICS dose, and procalcitonin can predict when antibiotic treatment can be stopped against an infectious asthma exacerbation. This is valuable, as ICS is a cornerstone in asthma treatment and overuse of antibiotics poses a huge challenge worldwide. However, there is a substantial gap in biomarkers predictive of disease remission and safe step-down of therapeutic treatment.

Asthma phenotypes may vary over time, and ideally, annual phenotyping may be required to update relevant treatment indication. Moreover, many asthma phenotypes, especially T2 low phenotypes, still lack relevant treatment options and not all treatments are endorsed by payers (e.g., treatment with anorectics and instructor guided exercise for obesity-associated asthma). Finally, current guidelines are not yet updated to endorse the use of biomarkers and precision medicine to treat asthma that is not severe.

## 5 Conclusion

Using biomarkers, it has become possible to identify subgroups of patients with severe asthma based on different phenotypes and endotypes. This process is ongoing and new findings are needed and constantly emerging, altering the way asthma is classified. Discovery of new biomarkers, improvement in the understanding of biomarkers associated with different types of asthma will and already is creating opportunities to offer targeted biologic treatments to patients with severe, uncontrolled asthma.

Some of these biomarkers may be used to fine-tune treatments and reduce corticosteroid use or dosage. Improvements in these guided treatment methods based on personalized medicine may result in simpler, cheaper treatment programmes that are more widely endorsed by clinicians, benefitting more patients with asthma.
